# Impact of alcohol-based hand-rub disinfection on bacterial bioburden on stethoscopes in a real-world clinical setting

**DOI:** 10.1017/ice.2022.54

**Published:** 2023-06

**Authors:** Alexandra A. Johnson, Bobby G. Warren, Deverick J. Anderson, Melissa D. Johnson, Isabella Gamez, Becky A. Smith

**Affiliations:** Duke Center for Antimicrobial Stewardship and Infection Prevention, Duke University Medical Center, Durham, North Carolina

## Abstract

In this randomized study, use of alcohol-based hand-rub disinfection significantly reduced bacterial bioburden of stethoscopes in routine clinical use. Prior cleaning of stethoscopes on the study day did not affect baseline contamination rates, which suggests that the efficacy of alcohol disinfection is short-lived and may need to be repeated between patients.

In the United States, ∼1 in 25 patients contract a healthcare-associated infection (HAI) each year, and ∼75,000 die in the hospital as a result of their HAI.^1^ HAIs contribute to increased length of hospitalization, morbidity, and mortality and ongoing work is needed to reduce the incidence of HAIs in hospital settings.

Stethoscopes are routinely colonized with pathogens and could serve as a vector for transmission.^2^ The Centers for Disease Control and Prevention (CDC) categorizes stethoscopes as noncritical patient-care items and recommends cleaning at least when visibly soiled and regularly, “such as after each use on each patient or once daily or once weekly.”^3^ This statement clearly leaves room for interpretation and may contribute to the lack of consistent stethoscope disinfection practices among clinicians. Common strategies used to clean stethoscopes pose certain barriers that prevent routine cleaning after every use. Previous research shows that the self-disinfection rate among clinicians is very low, which may be due to barriers such as time constraints.^4^ We sought to determine whether using readily available alcohol-based hand rub (ABHR) would effectively reduce bacterial bioburden on stethoscopes in a real-world setting.

## Methods

We performed a prospective, randomized study using stethoscopes from personnel working on the acute care floors of Duke University Hospital, a 979-bed, tertiary-care medical center in Durham, North Carolina. The study was approved by the Duke University Health System Institutional Review Board and had 2 arms: (1) no cleanser as a control and (2) active disinfection with isopropyl alcohol hand rub (ethyl alcohol 80% v/v, Alcare Extra Foaming Antiseptic Handrub, SC Johnson, Charlotte, NC). A waiver of informed consent was obtained because no protected health information was collected from study participants.

Physicians, medical students, physician assistants, nurse practitioners, and nurses and nursing students working on the inpatient wards of Duke University Hospital were randomly approached during rounds and asked to participate. Stethoscopes were randomized in blocks of 2 to control or intervention arms using a blocked randomization list generator.^5^ Participants randomized to intervention provided their stethoscopes to the study team, who then disinfected stethoscopes by applying 2 pumps of ABHR to their gloved hands and then applied it to the tubing, bell, and diaphragm. Stethoscopes were cultured after the ABHR completely dried (∼1 minute). Control stethoscopes were cultured directly with no intervention. Cultures were obtained from 80 stethoscopes over a period of 5 consecutive days, Monday through Friday in October 2019.

Cultures of the tubing and the bell and diaphragm for all stethoscopes were obtained with premoistened cellulose sponges (Whirl-Pak Premoistened Speci-Sponge Bags, Nasco, Modesto, CA). Sponges were combined with 45 mL 1% Tween20-PBS and mixed in the Seward Stomacher (Seward Limited, West Sussex, UK). The homogenate was centrifuged, and all but ∼5 mL of the supernatant was discarded. Samples were plated on sheep-blood agar and selective media for clinically important pathogens including *S. aureus*, *Enterococcus* spp, and gram-negative bacteria. Colony-forming unit (CFU) counts were determined by counting the number of colonies on each plate and using dilution calculations to calculate the CFU of the original ∼5 mL homogenate.

The following information was collected from each participant: hospital ward or service at the time of sampling; job role on the clinical team; age; sex; usual stethoscope cleaning frequency; and when they last cleaned their stethoscope (if known).

The sample size for the study was ∼80 stethoscopes, with 40 in each group. Based on previous studies, if there was a mean total bioburden of 60 CFU in the control group (SD, ±85) and 5 CFU in the ABHR, with a 2-sided α of 0.05 and a β of 0.2 (80% power), 74 stethoscopes would be needed. This count was rounded to 80 total stethoscopes.

A Wilcoxon rank-sum test was used to assess differences between the bacterial bioburden on control and intervention stethoscopes. We used JMP Pro SAS version 15.0 software (SAS Institute, Cary, NC) for all calculations. All statistical tests were 2-tailed, and *P* <.05 was considered statistically significant.

## Results

In total, 80 stethoscopes (40 disinfection and 40 control) were sampled from 46 physicians (MDs) and medical students (57.5%), 13 advanced practice providers (16.3%), and 21 nurses (RNs) and nursing students (26.3%). The median CFU count was significantly lower in the disinfection arm compared to the control: 106 CFU (IQR, 50–381) versus 3,320 CFU (IQR, 986–4,834; *P* < .0001). The effect was consistent across provider type, frequency of usual stethoscope cleaning, age, and status of pet ownership (Table 1). Of 80 healthcare workers, 60 (75%) indicated that they had not cleaned their stethoscope on the sampling day. Notably, median CFU was similar between the intervention group (395 CFU; IQR, 105–4,245) and the control group (457 CFU; IQR, 60–3,360), regardless of whether or not the participant had cleaned their stethoscope on the morning of the study day (*P* = 0.65, data not shown). Overall, 26 stethoscopes (33%) harbored a clinically important pathogen. The rates of presence of individual species of clinically important pathogen were lower but were not significantly different for stethoscopes that underwent disinfection versus controls: *S. aureus* (25% vs 32.5%), *Enterococcus* (2.5% vs 10%), and GNB (2.5% vs 5%).

**Table 1.** Analysis of Bacterial Bioburden on Stethoscopes that Underwent Alcohol Disinfection Versus Controls

Variable	Alcohol Disinfection (N = 40), Median CFU (IQR)	Control (N = 40), Median CFU (IQR)	*P* Value (Wilcoxon)
Total bioburden	106 (50–381)	3,320 (986–4,834)	<.0001
**Provider type**			
MD/Med student (n = 46)	162 (0–434)	2,820 (1,088–3,724)	<.0001
PA/NP (n = 13)	53 (0–420)	2,856 (774–7,740)	.0081
RN/Nursing student (n = 21)	82 (53–222)	4,592 (420–15,000)	.0014
**Frequency of usual stethoscope cleaning**			
Multiple times/day (n = 30)			
Daily (n = 18)	180 (60–348)	1,716 (240–12,155)	.0037
Weekly (n = 26)	264 (53–2,340)	3,572 (2376–7,272)	.0145
Less than weekly/Never (n = 6)	55 (0–138)	3,400 (1,221–4,216)	<.0001
	52 (0–660)	1,680 (1,200–3,400)	.0495
**Stethoscope cleaned on morning of study day**			
No (n = 60)	100 (0–420)	3,240 (944–4,410)	<.0001
Yes (n = 20)	108 (57–180)	4,020 (952–7,250)	.0014
**Stethoscopes positive for bacterial growth of a clinically important pathogen**	**No. (%)**	**No. (%)**	***P*** **Value (** ***X*** ^2^ **)**
*S. aureus*	10 (25)	13 (32.5)	.459
*Enterococcus*	1 (2.5)	4 (10)	FE .359
Gram–negative bacteria	1 (2.5)	2 (5)	FE 1.000

Note. IQR, interquartile range; FE, Fisher exact test.

## Discussion

Stethoscopes may serve as vectors for direct and indirect transmission of clinically important pathogens.^6^ Our study results demonstrate that using ABHR to clean stethoscopes after every use may be a practical and effective strategy to reduce overall bacterial contamination that can be easily incorporated into clinical workflow. Importantly, prior cleaning of stethoscopes on the study day did not seem to affect contamination rates, which suggests that alcohol foam disinfection is short-lived. As recently highlighted by Kalra et al,^4^ current guidelines need to be updated to include stethoscope disinfection between every patient. Our findings have implications beyond the present study, including disinfection of stethoscopes during the COVID-19 pandemic.^7^

Mehta et al^8^ evaluated the efficacy of ABHR (62% ethanol) compared to alcohol wipes (70% isopropyl) in disinfection of stethoscopes. Disinfection by either method was highly effective. Several other studies demonstrate similar efficacy of alcohol-based disinfection to reduce presence of a clinically important pathogen.^9^ These data suggest that use of the ABHR may be more practical because it can be completed in conjunction with hand hygiene.

This stethoscope bioburden study is the first to use the cellulose sponge method of sampling, which may be more effective at capturing bioburden on surfaces than other methods such as a swab or contact plates. This method allowed sampling of the tubing, bell, and diaphragm as opposed to some previous studies that cultured only the diaphragm. This difference likely contributed to our higher observed colony counts in both groups compared with those of previous studies.^2,8^

This study had several limitations. First, we were not powered to assess individual species of clinically important pathogen. Larger studies may be needed to determine the efficacy of ABHR at removing specific bacterial species from stethoscopes. Second, our study was conducted in a single academic medical center, and the baseline bioburden on stethoscopes in other settings may differ. Third, non–alcohol-based disinfectants were not included in the present study. In addition, we did not assess the presence of *C. difficile* on stethoscopes, which could also be clinically relevant and more resistant to ABHR disinfection. Finally, we did not evaluate the long-term impact of repeated alcohol exposure on stethoscope function.

In conclusion, in our prospective randomized study, stethoscopes were frequently contaminated with clinically important pathogens, and ABHR significantly reduced overall bacterial bioburden on stethoscopes. ABHR is a convenient and effective method of disinfecting stethoscopes. Additional education and interventions should be implemented to increase healthcare personnel awareness about methods and need for stethoscope disinfection after each use, similar to other nondedicated patient-care equipment.
